# Pituitary Apoplexy: An Uncommon Cause of Postpartum Headache

**DOI:** 10.7759/cureus.93658

**Published:** 2025-10-01

**Authors:** Maria Inês Silva, Sara Cabete, Diogo Brandão Neves, Cláudia Amaral, Gonçalo Madureira, Hermínia Cabido

**Affiliations:** 1 Anesthesiology, Intensive Care, and Emergency, Unidade Local de Saúde de Santo António, Porto, PRT; 2 Endocrinology, Unidade Local de Saúde de Santo António, Porto, PRT; 3 Neuroradiology, Unidade Local de Saúde de Santo António, Porto, PRT

**Keywords:** cranial nerve palsy, pituitary apoplexy, pituitary neuroendocrine tumor, post-dural puncture headache, postpartum headache, puerperium

## Abstract

When a postpartum headache follows a dural puncture, the diagnosis of post-dural puncture headache (PDPH) is usually considered; however, anesthesiologists must also recognize findings that warrant urgent specialized evaluation. Pituitary apoplexy (PA) is a rare but potentially life-threatening complication, and pregnancy itself is a recognized risk factor. The diagnosis should be suspected in the presence of a sudden-onset, severe headache, usually retro-orbital and worsening with time, visual disturbances, and cranial nerve palsy, especially in patients with additional risk factors such as a known pituitary neuroendocrine tumor. We report a case of postpartum PA in a woman with a pre-existing pituitary macroadenoma, who initially presented with severe headache attributed to PDPH. The headache progressively worsened despite therapy directed at PDPH, and she subsequently developed bradycardia and left oculomotor palsy. Imaging confirmed PA, and she was managed with intravenous hydrocortisone and transsphenoidal sellar decompression, followed by long-term endocrine replacement therapy. This case highlights the importance of considering this uncommon but critical condition in the differential diagnosis of postpartum headache in anesthetic practice. It also emphasizes the need to recognize red flags, such as a change in the nature or severity of a headache or the onset of focal neurological deficits, which should prompt urgent multidisciplinary evaluation and appropriate neuroimaging.

## Introduction

Headaches are a common complaint during the postpartum period, affecting up to one-third of women within the first week after childbirth. While most are benign primary headaches, such as migraine or tension-type headache, a significant proportion may result from secondary causes [1]. Secondary postpartum headaches include those of vascular or hypertensive origin, infection, space-occupying lesions, and complications of neuraxial procedures such as dural puncture. Distinguishing between primary and secondary causes is crucial, as delayed recognition of secondary headaches can lead to substantial morbidity and mortality [1,2]. When a headache occurs after a dural puncture, the diagnosis of post-dural puncture headache (PDPH) is often suspected [1]. PDPH is a frequent complication of spinal and combined spinal-epidural (CSE) anesthesia, particularly in obstetric practice, and its clinical presentation is typically diagnostic [3]. However, it is essential always to consider other potential causes, as a thorough differential diagnosis is critical [1] for the prompt identification of any secondary causes that may be life-threatening or significantly disabling. One rare but potentially life-threatening cause of postpartum headache is pituitary apoplexy (PA). This condition is considered a neuroendocrine emergency, requiring prompt diagnosis and management. Reported cases remain scarce, with available data limited to isolated case reports and small case series [4]. Anesthetists, who frequently assess women with postpartum headaches, must therefore be familiar with the possible differential diagnoses and able to recognize clinical features that warrant urgent referral for specialist evaluation. Prompt recognition of red flags such as sudden severe headache, visual disturbances, or cranial nerve palsies enables timely investigation and intervention, preventing potentially devastating endocrine and neurological complications [1,2]. We report a case of postpartum PA to highlight the importance of maintaining a broad differential diagnosis of postpartum headache in anesthetic practice.

## Case presentation

At 36 weeks and one day of gestation, a 31-year-old primigravida underwent an elective caesarean section due to a twin pregnancy complicated by intrauterine growth restriction. Her past medical history was notable for a non-functioning pituitary neuroendocrine tumor (PitNET, formerly known as pituitary adenoma), measuring 16.5×15×12.5 mm (macroadenoma). It was associated with a mild elevation in prolactin, likely due to the stalk effect, and was diagnosed during an infertility work-up. Treatment with cabergoline was initiated but discontinued following confirmation of pregnancy, which was achieved after ovulation induction.

Earlier in the pregnancy, at 15 weeks and six days of gestation, she presented with a one-week history of an isolated, pulsatile, right-sided hemicranial headache of moderate intensity. Neurological and ophthalmological evaluations were unremarkable. A follow-up pituitary MRI performed a few days later showed no significant change in the tumor. There was no subsequent neurological or endocrinological follow-up during the remainder of the pregnancy.

For the surgical procedure, CSE anesthesia using a dedicated kit was successfully performed on the first attempt. Apart from a brief initial hypotensive episode corrected with 10 mg of ephedrine, the patient remained hemodynamically stable. The surgery was uneventful. Postoperative analgesia was provided via an epidural catheter (morphine and ropivacaine boluses) along with systemic analgesics. Breastfeeding was successfully initiated in the immediate postpartum period.

At approximately 24 hours postpartum, the patient developed a sudden, severe bifrontal headache that worsened in the upright position and was accompanied by photophobia and nausea. The headache was unresponsive to systemic analgesics. Following anesthetic evaluation, a diagnosis of PDPH was made, and a sphenopalatine ganglion block (SPGB) was performed, resulting in significant pain relief. Analgesia and antiemetics were optimized, and caffeine therapy was initiated.

On postpartum day 2, the headache worsened, becoming pulsatile and persistent even in the supine position. The patient also experienced nausea, photophobia, and phonophobia and became bedridden. A second SPGB was performed, without clinical improvement. Several hours later, an epidural blood patch (EBP) was performed, which also failed to relieve symptoms.

In the early morning hours of postpartum day 3, new-onset bradycardia (heart rate 39-50 bpm) with normotension was documented, and the patient reported diplopia. Laboratory evaluation, including complete blood count, biochemical profile, and coagulation studies, was unremarkable. A few hours later, due to persistent, progressively worsening, severe, and refractory headache together with accompanying alarming symptoms, the patient was referred for urgent neurological consultation. Examination revealed an incomplete left oculomotor nerve palsy, characterized by mild ptosis, anisocoria (left > right) with preserved light reactivity, and marked limitation of ocular adduction, supraversion, and infraversion. Fundoscopic examination, performed by an ophthalmologist, was normal. Non-enhanced and venous phase computed tomography (CT) showed no evidence of cerebral venous thrombosis. However, it revealed enlargement of the known macroadenoma with hyperattenuating intratumoural components suggestive of hemorrhage, consistent with PA. Repeat full blood count, serum electrolytes, renal and liver function tests, and coagulation studies were normal.

An endocrinology evaluation was also performed. Given the high suspicion of PA, empiric intravenous hydrocortisone therapy was initiated, with a 100 mg bolus administered at 17:00. Hormonal assays were performed prior to hydrocortisone administration and repeated the following morning; the results are summarized in Table 1. Interpretation of values in the immediate post-partum period is challenging, yet the laboratory findings were suggestive of central adrenal insufficiency and central hypothyroidism, with no evidence of other pituitary hormone deficits. Following repeat hormonal assessment, hydrocortisone therapy was recommenced at a dose of 50 mg every six hours.

**Table 1:** Endocrine laboratory results

Parameter	Result		Normal range
	Postpartum day 3	Postpartum day 4	
	16:50 (time of blood draw)	09:00 (time of blood draw)*	
Adrenocorticotropic hormone (pg/mL)	-	12.9	Morning: 9-52
Cortisol (μg/dL)	1.8	13.1	Morning: 6.2-19.4
			Evening: 2.3-11.9
Growth hormone (ng/mL)	5.43	2.59	0.06-10.00
Somatomedin C (ng/mL)	152	-	71.2-234
Prolactin (ng/mL)	20.3	22.6	4.79-23.3
Thyroid-stimulating hormone (μIU/mL)	1.07	0.86	0.30-3.94
Free T4 (ng/dL)	0.65	0.65	0.95-1.57
Luteinizing hormone (mIU/mL)	<0.3	-	Follicular: 2.4-12.6
			Ovulatory: 14.0-95.6
			Luteal: 1.0-11.4
Follicle-stimulating hormone (mIU/mL)	<0.3	-	Follicular: 3.5-12.5
			Ovulatory: 4.7-21.5
			Luteal: 1.7-7.7
*Last hydrocortisone bolus was at 17:00 on the previous day			

**Figure 1:**
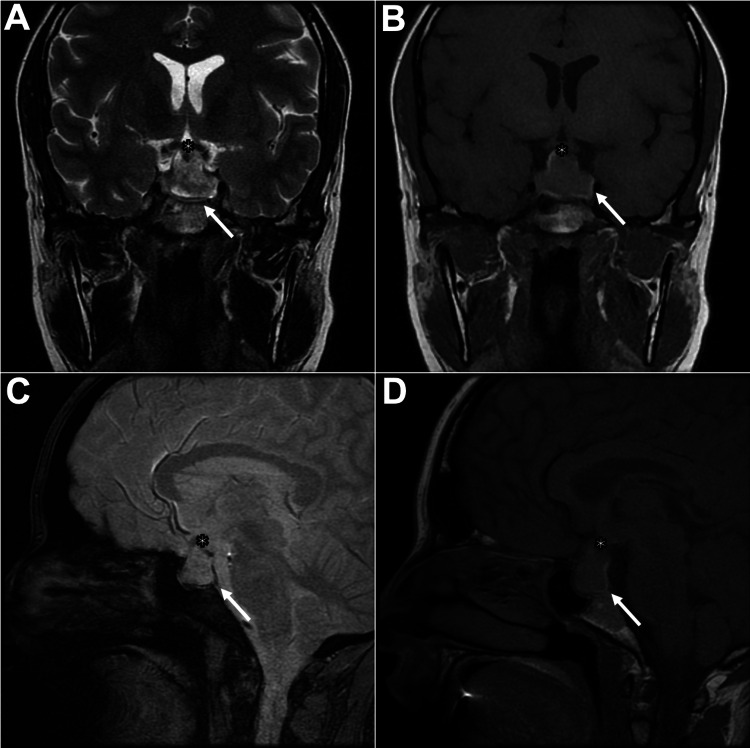
MRI on postpartum day 3 demonstrating hemorrhage within a pituitary macroadenoma, confirming apoplexy Coronal T2-weighted (A), coronal T1-weighted (B), sagittal T2*-weighted (C), and sagittal T1-weighted (D) images demonstrate interval growth of the pituitary lesion (23×25×13.5 mm) with marked suprasellar extension, contacting and displacing the optic chiasm superiorly (asterisk). The lesion also shows slight parasellar extension, abutting the medial wall of the cavernous sinuses. Increased internal heterogeneity is observed, with a peripheral rim that is hyperintense on T1-weighted (B, D) and hypointense on T2 (A) and T2*-weighted (C) images, consistent with acute hemorrhagic products (arrow). Findings are compatible with pituitary apoplexy.

A brain MRI performed shortly afterward confirmed the diagnosis of PA (Figure 1).

Following neurosurgical consultation, surgical intervention was planned.

On postpartum day 4, the patient underwent endoscopic transsphenoidal sellar decompression with hematoma drainage and partial tumor resection. The procedure was uneventful. Postoperatively, she was managed in the neurosurgery ward and was treated with hydrocortisone, levothyroxine, and desmopressin (only a single dose was administered). Symptoms resolved within 48 hours. She was discharged on a supraphysiologic, fixed-dose regimen of hydrocortisone (10+5+5 mg) and levothyroxine (50 µg daily), with desmopressin prescribed on an as-needed basis, until the endocrinology appointment. Outpatient follow-up with neurosurgery was also scheduled.

Histopathological analysis revealed findings consistent with PA within a PitNET, although the tumor cell lineage could not be determined.

During endocrinology outpatient follow-up, the diagnosis of adrenal insufficiency and central hypothyroidism was confirmed, along with the identification of deficits in the gonadotrophic and somatotrophic axes. She remains on hormonal replacement therapy with hydrocortisone, levothyroxine, and estrogen. As there was no recovery of the somatotrophic axis after one year, recombinant human growth hormone therapy was initiated, resulting in a marked improvement in quality of life.

**Figure 2:**
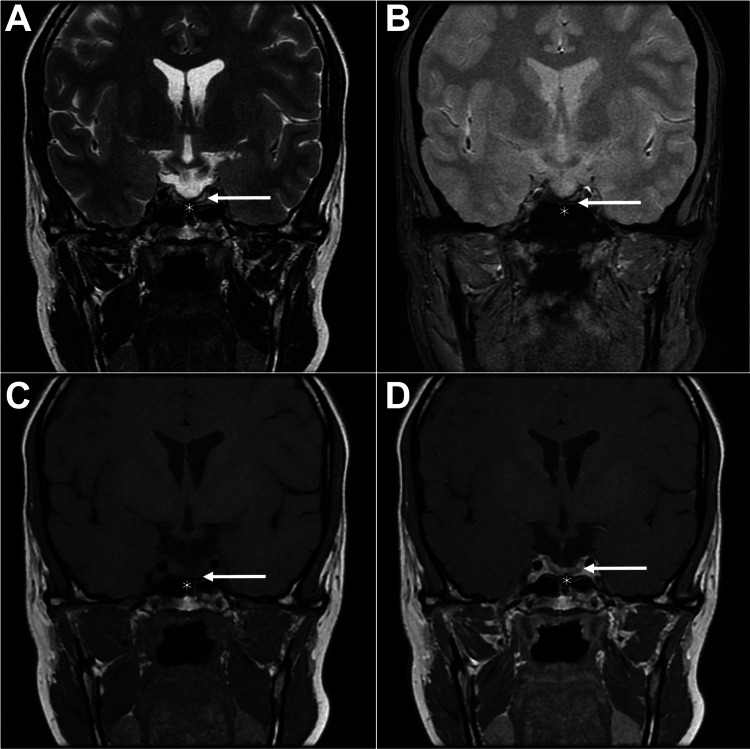
One-year postoperative follow-up MRI after transsphenoidal surgery Coronal T2-weighted (A), coronal T2*-weighted (B), coronal T1-weighted (C), and post-contrast coronal T1-weighted (D) images demonstrate postoperative changes consistent with a transsphenoidal approach, including sellar floor dehiscence (asterisk). The sella is filled with cerebrospinal fluid-equivalent signal intensity. A thin linear remnant of pituitary tissue is evident along the inferior margin (arrow). No residual tumor is identified. The optic chiasm appears normal.

A follow-up MRI showed no evidence of residual or recurrent tumor. The sella turcica was largely filled with cerebrospinal fluid, with the remaining normal pituitary tissue (thinned) adjacent to the sella floor (Figure 2).

**Figure 3:**
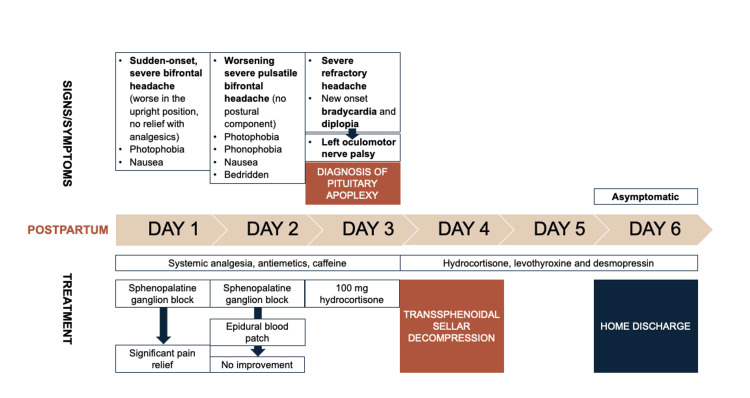
Chronology of events described in the reported case

Figure 3 illustrates the chronology of events described in the case report.

Written informed consent was obtained from the patient for publication of this case report and accompanying images. 

## Discussion

According to current clinical practice guidelines, PDPH should be suspected if headache or neurological symptoms, which may be relieved when lying flat, occur within five days of a neuraxial procedure [5]. The diagnosis of PDPH is based on the clinical presentation and a detailed history and physical examination [6]. The typical presentation is a bilateral frontal or occipital headache with a postural component. Associated symptoms may include nausea and vomiting, neck and shoulder stiffness, back or intrascapular pain, visual disturbances (e.g., photophobia, diplopia, and blurred vision), auditory symptoms (e.g., tinnitus and hypoacusis), and vertigo or dizziness. The physical examination is usually unremarkable. In atypical cases, cranial nerve palsies may be observed (III-VIII). The abducens and facial nerves are the most affected [7].

Given the patient's clinical presentation and anesthetic history, PDPH was diagnosed despite an uncomplicated CSE technique. Evidence suggests that all neuraxial techniques have similar PDPH risk profiles [5].

However, even when the diagnosis of PDPH appears obvious, as in our case, it is essential always to rule out alternative diagnoses. In addition to evaluating neuraxial techniques for labor and delivery, it is essential to conduct a comprehensive history, with particular attention to previous headaches, relevant obstetric and medical history, and risk factors for postpartum headache. The neurological examination should assess for features of headaches that are included in the differential diagnosis [1]. Any variation in headache severity (e.g., worsening of a pre-existing headache) or nature (e.g., transition from orthostatic to non-orthostatic in patients with a diagnosis of PDPH), features suggestive of intracranial hypertension, association with elevated blood pressure, new-onset severe headache, thunderclap headache, altered level of consciousness, seizures, visual disturbances, focal neurological deficits, or fever, warrant urgent neuroimaging and prompt consultation with an appropriate specialist to evaluate for alternative diagnoses [1,3,5,8]. The same clinical approach should be applied to PDPH patients who exhibit worsening symptoms despite receiving an EBP [5].

In this case, diagnostic reassessment was likely delayed because the initial symptoms appeared typical of PDPH. As PDPH is the most frequent cause of postpartum headache in our anesthetic practice, alternative diagnoses were not promptly considered. Additionally, the patient’s history of a pituitary macroadenoma did not prompt suspicion of related complications. 

The evolving nature of the headache, progressively worsening and losing its postural component, was not immediately recognized as an indicator of alternative pathology. Only after the complete failure of the EBP and the emergence of diplopia and bradycardia was the patient referred for a neurology consultation.

The specialized investigation ultimately led to the diagnosis of PA. Its prevalence in the postpartum period is unknown. In a case series, prevalence was estimated at one per 10,000 term pregnancies. Furthermore, in the associated 54-year literature review, only 33 reported cases of PA during pregnancy were found [9].

Apoplexy results from hemorrhage and/or infarction of the pituitary gland in the setting of a PitNET or a physiologically enlarged pituitary gland. Most cases involve an underlying PitNET, particularly nonfunctioning tumors or prolactinomas, with a higher risk in macroadenomas. Pregnancy is a recognized risk factor as hormonal changes lead to an enlargement of the pituitary gland by up to 120% of its original volume, peaking around the third day postpartum. Other risk factors associated with PA include acute changes in blood pressure, systemic hypertension, diabetes mellitus, coagulation disorders, dopamine agonist treatment, and pituitary stimulation tests [8-10].

During pregnancy, women with a known PitNET, particularly macroadenomas, should undergo regular clinical follow-up by an endocrinologist. If tumor progression or apoplexy is suspected, a neuro-ophthalmologic examination and non-contrasted MRI are recommended [11]. 

Hemorrhage and/or necrosis raise intrasellar pressure, leading to varying degrees of compression of adjacent structures and producing diverse manifestations: increased intracranial pressure from mass effect, cranial nerve involvement from compression, and hypopituitarism from pituitary parenchymal injury. The clinical course depends on the rate of onset of infarction or hemorrhage, as well as the volume of hemorrhage, and the presentation may be acute or subacute [10].

The most common symptom is a sudden-onset severe headache that can be thunderclap in nature, often retro-orbital. Ocular visual impairment is the next most frequent manifestation and is due to optic chiasm or optic nerve involvement (e.g., hemianopsia, unilateral vision loss, blurred vision, and transient loss of vision) [3,4]. Other associated features may include nausea, vomiting, meningism (e.g., photophobia and neck stiffness), and altered mental status ranging from mild encephalopathy to coma. Cranial nerve involvement (III, IV, V1, V2, and VI) may occur due to cavernous sinus involvement. Most frequently affects the third cranial nerve [3,12].

This condition is a medical emergency due to the risk of acute pituitary insufficiency. Pituitary hormone deficiencies may develop quickly but are not always present. The deficiencies are often multiple, with adrenocorticotropic hormone insufficiency being the most critical and potentially fatal complication, leading to acute adrenal insufficiency with hyponatremia, hypotension, hypoglycemia, and altered mental status. Other frequently affected hormones are growth hormone, thyroid hormone, and gonadotropins. Diabetes insipidus may also occur [10].

Diagnosis relies on prompt neuroimaging, with MRI regarded as the gold standard. However, in neurological emergencies, CT is frequently the first-line imaging modality due to its wide availability, rapid acquisition, and diagnostic accuracy for emergent intracranial conditions. This also applies in pregnancy, as fetal radiation exposure from a head CT is considered negligible [3].

Urgent and comprehensive assessment of pituitary hormones is essential, noting that the usual diagnostic criteria for deficiencies outside pregnancy do not apply during gestation and early puerperium [4,10].

Optimal treatment and timing of intervention are still under debate. Thus, the therapeutic approach to patients with PA tends to be empirically individualized. Initial management includes high-dose corticosteroids, hemodynamic stabilization, fluid and electrolyte correction, appropriate hormonal replacement, and specific treatment for the underlying causes of PA. High-dose corticosteroids are typically initiated as empiric replacement therapy due to the high likelihood of central adrenal insufficiency. Their anti-inflammatory and antiedematous effects are also beneficial. Thyroid hormone replacement should always be preceded by assessment and, if necessary, replacement of adrenal insufficiency, as it may precipitate an adrenal crisis. Close monitoring of visual acuity, field loss, ocular motility, and level of consciousness is essential [10].

Surgery is generally reserved for seriously ill patients with significant neuro-ophthalmic signs, reduced level of consciousness, or lack of response to conservative treatment. After surgery, improvement in visual deficits is common, usually occurring in the immediate postoperative period. Approximately 50% of patients recover pituitary function and can discontinue pituitary hormone replacement therapy; therefore, reassessment of endocrine status should be performed [13].

An experienced multidisciplinary team should manage patients with PA, and the clinical outcomes are closely related to the team's skills and expertise [10]. In the present case, coordinated care was provided by specialists in anesthesiology, neurology, ophthalmology, endocrinology, and neurosurgery.

## Conclusions

Even in the presence of a known dural puncture and likelihood of PDPH, anesthesiologists must remain alert to alternative diagnoses, especially in the presence of red flag features. Changes in the nature or severity of the headache, such as progression from postural to non-postural, worsening intensity, or the onset of new neurological deficits, should prompt urgent and multidisciplinary reassessment. A thorough differential diagnosis is essential to prevent delayed recognition of potentially life-threatening conditions.

This case highlights the importance of considering PA in the differential diagnosis of postpartum headache, especially in patients with additional risk factors such as known PitNETs. Its signs and symptoms may overlap with and mimic other neurological conditions. A high index of suspicion is therefore crucial, particularly in the presence of a sudden-onset severe headache, usually retro-orbital and worsening with time, along with visual disturbances and cranial nerve palsy. Early recognition enables timely neuroimaging and treatment to avoid endocrine and neurological complications, while delayed or missed diagnosis may result in considerable morbidity and mortality.

## References

[1] Janvier AS, Russell R (2022). Postpartum headache - diagnosis and treatment. BJA Educ.

[2] Sabharwal A, Stocks GM (2011). Postpartum headache: diagnosis and management. Contin Educ Anaesth Crit Care Pain.

[3] O'Neal MA (2017). Headaches complicating pregnancy and the postpartum period. Pract Neurol.

[4] Pop LG, Ilian A, Georgescu T, Bacalbasa N, Balescu I, Toader OD (2022). Pituitary adenoma apoplexy in pregnancy: case report and literature review. Exp Ther Med.

[5] Uppal V, Russell R, Sondekoppam RV (2024). Evidence-based clinical practice guidelines on postdural puncture headache: a consensus report from a multisociety international working group. Reg Anesth Pain Med.

[6] (2025). American Society of Anesthesiologists. Statement on post-dural puncture headache management. https://www.asahq.org/standards-and-practice-parameters/statement-on-post-dural-puncture-headache-management.

[7] Chambers DJ, Bhatia K (2017). Cranial nerve palsy following central neuraxial block in obstetrics - a review of the literature and analysis of 43 case reports. Int J Obstet Anesth.

[8] Khoromi S (2023). Secondary headaches in pregnancy and the puerperium. Front Neurol.

[9] Grand'Maison S, Weber F, Bédard MJ, Mahone M, Godbout A (2015). Pituitary apoplexy in pregnancy: a case series and literature review. Obstet Med.

[10] Iglesias P (2024). Pituitary apoplexy: an updated review. J Clin Med.

[11] Luger A, Broersen LH, Biermasz NR (2021). ESE Clinical Practice Guideline on functioning and nonfunctioning pituitary adenomas in pregnancy. Eur J Endocrinol.

[12] Gheorghe AM, Trandafir AI, Stanciu M, Popa FL, Nistor C, Carsote M (2023). Challenges of pituitary apoplexy in pregnancy. J Clin Med.

[13] Arafah BM, Harrington JF, Madhoun ZT, Selman WR (1990). Improvement of pituitary function after surgical decompression for pituitary tumor apoplexy. J Clin Endocrinol Metab.

